# Splenic Hematoma Mimicking Angiosarcoma: A Case Report

**DOI:** 10.1155/2012/183458

**Published:** 2012-08-08

**Authors:** Seckin Akkucuk, Akin Aydogan, Hasan Gokce, Ramazan Davran, Murat Karcioglu

**Affiliations:** ^1^Department of General Surgery, Faculty of Medicine, Mustafa Kemal University, Hatay, 31100 Serinyol, Turkey; ^2^Department of Pathology, Faculty of Medicine, Mustafa Kemal University, Hatay, 31100 Serinyol, Turkey; ^3^Department of Radiology, Faculty of Medicine, Mustafa Kemal University, Hatay, 31100 Serinyol, Turkey; ^4^Department of Anesthesiology, Faculty of Medicine, Mustafa Kemal University, Hatay, 31100 Serinyol, Turkey

## Abstract

*Introduction*. Splenic hematomas usually occur after blunt abdominal trauma. Most of the subcapsular hematomas will be resolved and reabsorbed spontaneously. However in rare cases, some of them organize and form calcified splenic masses. Angiosarcoma is an uncommon primary tumor of the spleen. Splenic angiosarcoma behaves extremely aggressive and has poor prognosis. *Case Presentation*. We report a forty-nine-year-old white male with organized splenic hematoma due to traffic accident mimicking splenic angiosarcoma. *Conclusion*. Both angiosarcoma and splenic organized hematoma have nonspecific symptoms and clinical findings. Because of the risk of hemorrhage and rupture, fine-needle biopsy should not be preferred. In case of splenic masses, excision and spleen-conserving surgery or total splenectomy should be performed.

## 1. Introduction

The spleen is one of the most injured organs after blunt abdominal trauma [1, 2]. Of the splenic injuries due to blunt abdominal trauma, 40–70% can be followed without surgical intervention [1–3]. A majority of splenic hematoma of nonoperated patients can resolve within 2 or 3 months spontaneously. At the other hand, the hematoma, rarely, organizes and at last calcifies [1]. 

Primary angiosarcoma of spleen is a rare malignancy [4–6]. It is difficult to diagnose the tumor since its symptoms are not specific. Although imaging technics are important for the diagnosis, they rarely give differential information about the nature of the splenic masses [5, 6]. 

We reported a case of splenic mass in a patient with previous blunt abdominal trauma that was reported as splenic angiosarcoma at magnetic resonance imaging. We planned laparotomy for a definitive diagnosis and also for treatment. The histopathological examination revealed it as splenic hematoma. 

## 2. Case Presentation

**Figure 1:**
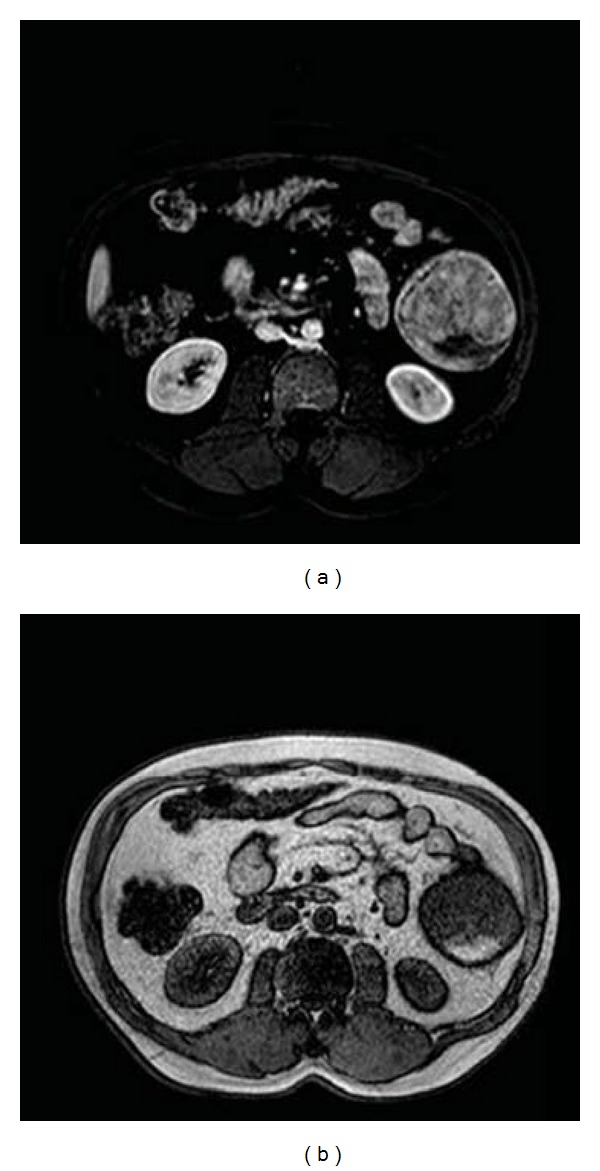
(a) Intense contrast enhancement of splenic mass. No enhancement detected at the inferior side suggesting bleeding. (b) MRI: regular-bordered mass with heterogenic hyperintense area at inferior pole on axial T1 weight pulse sequence.

**Figure 2:**
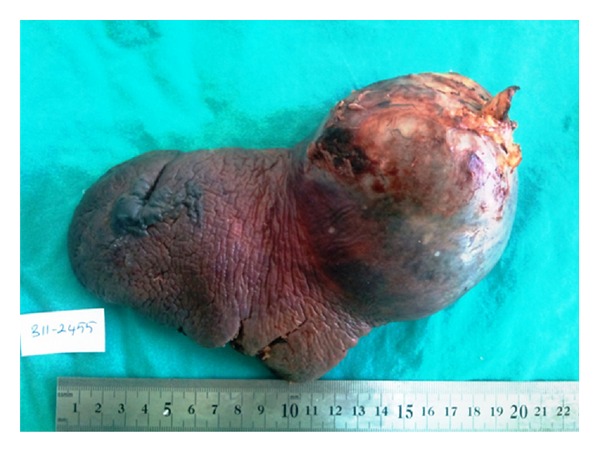
Eight centimeter mass inferior to the spleen.

**Figure 3:**
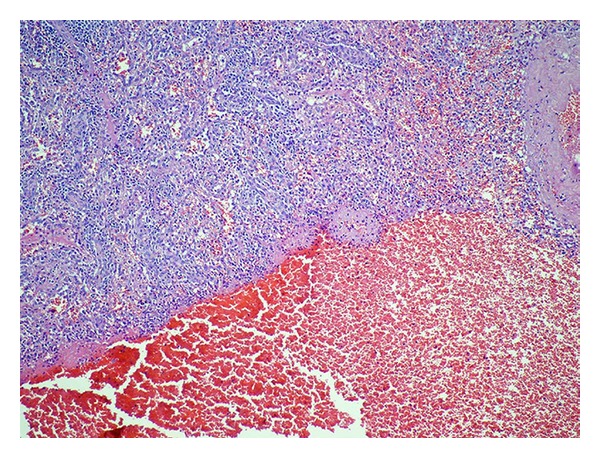
Hematoma including the accumulation of erythrocyte without epithelial lining in the lower half of picture.

A forty-nine-year-old male with bilateral hypocondrial and epigastric nonspecific abdominal pain, sometimes with nausea and vomiting, was admitted to our hospital. He had a medical history of hospitalization and medical treatment due to car accident 18 months ago. Clinical examination was unremarkable. His complete blood count and biochemical tests were between normal ranges. Abdominal ultrasound (USG) images revealed 8 mm stone with normal wall thickness in gallbladder and a heterogenic complex mass of 8 cm in diameter at the inferior part of the spleen. Then magnetic resonance imaging (MRI) was performed to the patient. It revealed the same findings as USG, an 8 mm single stone in gallbladder and 80 mm heterogenic hyperintense on T2 weight pulse sequence, and heterogenic hypointense on T1 weight pulse sequence mass in spleen. The mass showed heterogenic enhancement after contrast agent injection, and its margins were regular (Figure 1). The USG and MRI could not differentiate its nature, if it was benign or malign (organized hematoma or angiosarcoma). We planned to perform cholecystectomy and splenectomy with mass excision. The laparotomy started with higher median incision. During exploration an 8 cm mass on the lower pole of spleen at anterolateral position was detected (Figure 2). Finally, cholecystectomy and splenectomy including mass excision were performed. The histopathological examination of the mass was reported as organized hematoma (Figure 3). The patient was discharged at postoperative 5th day without any complication.

## 3. Discussion 

The splenic masses are quite rare and may be benign, malign, granulomatous, and even inflammatory or traumatic pseudotumors [7]. The most seen primary malignant tumor of spleen is angiosarcoma. Angiosarcoma is an uncommon aggressive tumor with poor prognosis [4–6]. A review of medline showed no more than 100 cases of primary splenic angiosarcoma. Some of them can be diagnosed during autopsy [6]. Although it is not obvious, it is more common in men in the fourth decade [4]. Metastases occur in about 80% of patients and most common sites are liver, lung, lymph nodes, and bone. Metastases usually occur via hematogenous dissemination. The mean overall survival after diagnosis is about 10.3–14.4 months [6]. Clinical symptoms of splenic angiosarcoma are not specific, so diagnosis may be harder due to symptoms. The most common presentation is the upper abdominal pain (75–80%), splenomegaly, fatigue, and weakness [4–6]. USG, MRI, abdominal computed tomography may reveal an ill-defined heterogeneous splenic mass with areas of necrosis, and this hardens a definitive diagnosis [4–6, 8]. In our case, USG and MRI detected a heterogeneous splenic mass suggesting hematoma or angiosarcoma. Also fine-needle biopsy from the mass is not advised due to the high risk of hemorrhage and rupture [6].

During laparotomy, the macroscopic view of the mass may vary from nodular structure to enlarged mass with areas of hemorrhage and necrosis [4]. The only therapeutic method in splenic angiosarcoma is splenectomy [5, 6]. There are poor data about the efficacy of chemotherapy because of the small number of cases [6]. 

Sometimes splenic angiosarcoma may be presented as splenic rupture (32%). This is usually fatal, and half of these cases can be diagnosed on postmortem examination [5, 6]. 

The spleen is one of the most vulnerable organs after blunt abdominal trauma. Almost two-thirds of blunt abdominal traumas causing splenic injuries occur due to road traffic accident. Subcapsular hematomas are seen in 37% of splenic injuries [1, 2]. Forty to seventy percent of these hematomas can be managed nonoperatively [1–3]. Most of the subcapsular hematomas are resolved and then reabsorbed spontaneously within 1-2 months, but some hematomas lead to delayed rupture. Rarely, a little number of the subcapsular hematomas can be organized and become calcified splenic masses [1, 2]. Noncalcified hematomas that did not resolve spontaneously can be drained by percutaneous aspiration methods [1]. Otherwise, partial splenectomy and resection of hematoma should be performed in terms of organized-calcified hematomas. However, considering the reduced function of remnant spleen and the fragility of the spleen in the elderly, and the safety of surgery for senile patient, splenectomy should be the preferred choice [1].

Subcapsular hematomas presenting as splenic masses are usually asymptomatic. Sometimes nonspecific abdominal pain and palpable mass may be the symptoms. During clinical examination, physicians rarely can palpate the splenic hematomas [1–3]. Radiologic differentiation of organized subcapsular hematoma from other malign or benign splenic masses usually cannot be possible [2]. 

Our patient's presenting symptom was the abdominal pain as seen in splenic angiosarcoma or splenic subcapsular hematoma. Physical examination findings were not diagnostic except suspicious left upper quadrant mass. Anemia can be seen in 80% of splenic angiosarcoma patients, but it is not a diagnostic criterion alone [4]. In our case, complete blood counting results were in normal ranges. Abdominal USG and MRI both defined a splenic mass of 80 mm, but could not reveal its nature. 

Splenic masses have disadvantages in diagnosis because of infrequency and inadequate investigation with laboratory and radiologic imaging [2, 5]. On the other hand, the presence of blunt abdominal trauma history should suggest organized subcapsular hematoma. However, organized hematomas are not suitable for percutaneous approach and cause permanent pain, so they require surgical excision. In cases of benign splenic masses like hematoma, if the remnant splenic parenchymal tissue is enough, and the spleen is not fragile, the preferred surgical choice should be one of the spleen-conserving methods (like excision of hematoma, partial splenectomy, etc.) [1]. In our case, we avoided biopsy because of high risk of hemorrhage and rupture. For this reason, we could not rule out angiosarcoma, and we preferred splenectomy rather than conserving methods. 

One of the serious complications of splenectomy is overwhelming postsplenectomy infection (OPSI). It is a rare but very dangerous situation. Spleen is the largest organ of lymphatic system and is one of the sites of antibody production. After splenectomy, patients become immune suppressed. The mortality rate due to fulminant sepsis is 4.4% in children and 0.9% in adults. Vaccine immunoprophylaxis against pneumonia, influenza, and meningitis is essential for asplenic patients. Particularly, pneumococcal vaccine is recommended at least 2 weeks before the splenectomy procedure. The vaccine should be repeated for every 5–10 years. Also patient education against OPSI will be very useful [9]. We vaccinated the patient 15 days before the operation. Also patient education is performed.

We recommend surgical removal and histopathological examination of the splenic masses, if there is suspicious about its nature. Blunt abdominal trauma history may suggest subcapsular hematoma in such cases. Spleen-conserving surgery in proper cases should be the appropriate choice. However, if there is doubt about the nature of the mass, splenectomy should be preferred.
